# Pharmacotherapy for bipolar disorder and concordance with treatment guidelines: survey of a general population sample referred to a tertiary care service

**DOI:** 10.1186/1471-244X-13-211

**Published:** 2013-08-13

**Authors:** Sabrina Paterniti, Jean-Claude Bisserbe

**Affiliations:** 1 Department of Psychiatry, University of Ottawa, Ottawa, ON, Canada; 2 Royal Ottawa Mental Health Centre, 1145 Carling Avenue, Ottawa, Ontario K1Z 7K4, Canada

**Keywords:** Bipolar disorder, Pharmacotherapy, Treatment, Guidelines

## Abstract

**Background:**

Many new approaches have been adopted for the treatment of bipolar disorder (BD) in the past few years, which strived to produce more positive outcomes. To enhance the quality of care, several guideline recommendations have been developed. For study purposes, we monitored the prescription of psychotropic drugs administered to bipolar patients who had been referred to tertiary care services, and assessed the degree to which treatment met specific guidelines.

**Methods:**

Between December 2006 and February 2009, we assessed 113 individuals suffering from BD who had been referred to the Royal Ottawa Mental Health Centre (ROMHC) Mood Disorders Program by physicians within the community, mostly general practitioners. The Structured Clinical Interview for DSM-IV-TR was used to assess diagnosis. The prescribed treatment was compared with specific Canadian guidelines (CANMAT, 2009). Univariate analyses and logistic regression were used to assess the contribution of demographic and clinical factors for concordance of treatment with guidelines.

**Results:**

Thirty-two subjects had BD type I (BD-I), and 81 subjects had BD type II (BD-II). All subjects with BD-I, and 90% of the BD-II group were given at least one psychotropic treatment. Lithium was more often prescribed for subjects with BD-I (62%) than those with BD-II (19%). Antidepressants were the most frequently prescribed class of psychotropics. Sixty-eight percent of subjects received treatment concordant with guidelines by medication and dose. The presence of a current hypomanic episode was independently associated with poorer concordance to guidelines. In more than half the cases, the inappropriate use of antidepressants was at the origin of the non concordance of treatment with respect to guidelines. Absence of psychotropic treatment in bipolar II patients and inadequate dosage of mood stabilizers were the two other main causes of non concordance with guidelines.

**Conclusions:**

The factors related to treatment not concordant with guidelines should be further explored to determine appropriate strategies in implementing the use of guidelines in clinical practice.

## Background

Bipolar disorder (BD) is a chronic disease with high risk of relapse. This disease also results in a high rate of suicidal mortality [1]. Many treatments have been made available in recent years to manage BD and to enhance positive outcome. To improve the quality of care, several guidelines have been developed with the help of evidence-based medicine and expert consensus meetings [2-9]. Given the rapid growth in the number of available medications, some of these guidelines have been updated [10-14]. The first guidelines which were published in 1997 by the The Canadian Network for Mood and Anxiety Treatments (CANMAT) [15] were updated in 2005 [16]. This update resulted in major modifications in its recommendations while projecting integrated elements of efficacy, effectiveness, and side effects. Further updates appeared in 2007 and 2009 [17,18]. CANMAT guidelines pointed out specific recommendations for bipolar II disorder (BD-II), which was neglected by most of the previous guidelines.

Two studies from the Texas Medication Algorithm Project underline the importance of enhanced adherence to clinical guidelines which can effectively improve the outcome of bipolar patients. Suppes et al. [19] assessed 141 bipolar patients treated in clinics where treatment guidelines were actively implemented in meetings with clinicians and in psychoeducational interventions with patients and families. They compared their outcome with that of 126 patients treated in a standard “as usual” protocol. The first group of patients showed greater sustained improvement at the initial and later stages with respect to overall severity of psychiatric symptoms, as well as in manic symptoms. For the same group of 141 bipolar patients, stricter adherence to treatment guideline recommendations was associated with greater reductions in depressive symptoms and overall psychiatric symptoms over time [20].

Despite the recent emphasis on evidence-based practice, guideline recommendations are not always implemented. Perlis [21] found that 34% of psychiatrists reported not having recourse to guidelines on a regular basis to treat BD, where only a very small percentage identified guidelines as their primary source of information. Similar results were obtained in two other studies conducted in France and Serbia, where 40% and 34% of psychiatrists, respectively, had claimed non-use of guidelines in the management of BD in daily practice [22,23].

The treatment of bipolar patients in concordance with guidelines varies widely throughout the studies, as shown in Table 1. Except for the low percentages found in three studies [24-26], concordance percentages ranged from 50% to 80%. Smith et al. [27] found that concordance of treatment was 52% in a naturalistic setting, with a percentage increasing to 75% after a one-year psychoeducational intervention aimed at implementing the guidelines. Bauer et al. [28] found a concordance of 50-60% at the index hospitalization with respect to 306 participants monitored through a collaborative care model; there was, however, a marked decrease after 2 years. Dennehy et al. [29] found high percentages of concordance with treatment guidelines among psychiatrists who had participated in extensive training based on published clinical practice guidelines.

**Table 1:** Studies of guideline concordance in bipolar disorder

	**Population**	**N**	**Guidelines**	**% Concordance**
Unutzer et al. 2000 [30]	Out-patients	1246 BD	Expert consensus panel	83%
Lim et al. 2001 [24]	In-patients	1421 BDI	Expert consensus panel	Manic with psychotic features: 38% without: 16%; Depressive with psychotic features: 31%; without: 17%
Simon et al. 2004 [31]	Out-patients	665 BDI + 239 BDII	Several guidelines	BDI: 65.4% BDII: 52.7%
Kilbourne et al. 2005 [32]	In- and out-patients	2316 BDI	APA and Department of Veterans Affairs	Bipolar specific MS: 74.6% Any MS or neuroleptics second generation: 84.4%
Farrelly et al. 2006 [33]	Out-patients	224 mood episodes in 84 BD I and II	British Association for Psychopharmacology	Manic: 81% Depressive: 64% Mixed: 62%
Dennehy et al. 2007 [29]	Out-patients	469 BDI 198 BDII	Several guidelines	Depressive: 83.4% Hypomanic/manic: 81.9% Mixed: 81.8%
Bush et al. 2007 [34]	In- and out-patients	2644 BDI	Several guidelines	67%
Arvilommi et al. 2007 [25]	In- and out-patients	BDI – 90 BDII- 101	Several guidelines	BDI: 55.6% BDII: 30.7%
Smith et al. 2008 [27]	In-patients	23 BD	Expert consensus panel	52% in naturalistic settings, increased to 75% after intervention
Bauer et al. 2009 [28]		306 (87% BDI)	Department of Veterans Affairs	50%-60% at index hospitalization in collaborative model; declined to 30%-40% after two years
Altinbas et al. 2011 [26]	Out-patients	263 depressive episodes in 142 patients (131 BDI)	Turkish Psychiatric Association Guidelines for Bipolar Disorder	Severity of the episode: Mild: 29.%; Moderate or severe without psychosis: 27.4%; severe with psychos: 87.5%

*BD* Bipolar Disorder, *BDI* Bipolar Disorder type I, *BDII* Bipolar Disorder type II, *MS* Mood Stabilizer, *APA* American Psychiatric Association.

Some factors were linked to better concordance with guidelines. Manic patients were more likely to receive concordant treatment than patients suffering from depressive or mixed episodes [33]. The presence of psychotic features was related to higher percentages of concordance with treatment guidelines [24,26]. Treatment setting influenced concordance to treatment guidelines, with in-patients more frequently receiving adequate treatment than out-patients [25,34]. Patients diagnosed with anxiety or depressive disorder before being diagnosed with BD were less likely to receive antimanic medications and more likely to receive antidepressants without any antimanic treatment [34]. In the STEP-BD study, earlier age at onset and administration of adequate pharmacotherapy at entry predicted those more likely to receive guideline-concordant care during new-onset mood episodes [29]. In the same STEP-BD study [31], bipolar I patients were more likely to receive adequate treatment as opposed to bipolar II patients. Arvilommi et al. [25] found that with the lack of clinical diagnosis of BD at entry, rapid cycling, polyphasic index episode, as well as depressive index phase, were independently associated with inadequate treatment. In a study assessing the treatment of veterans suffering from BD [32], race was not an associative factor in the administration of mood stabilizers.

To our knowledge, there has never been a study conducted on concordant treatment of bipolar patients in keeping with the CANMAT guidelines in Canada. The first object of our study was to examine the administration of psychotropic drugs among subjects diagnosed with BD and who had been referred to tertiary care services. The second objective was to assess concordance of prescribed treatments with Canadian guidelines. The third objective was to identify the clinical factors linked to poorer concordance of treatment with CANMAT guidelines.

## Methods

### Sample

The study “Prevalence, comorbidity and psychosocial risk factors: recurrent unipolar depression and bipolar disorder” was realized in the context of the Assessment and Treatment Clinic (ATC), an out-patient service established in 2006 within the framework of the Royal Ottawa Mental Health Centre (ROMHC) Mood Disorders Program. The ROMHC Mood Disorders Program provides specialized tertiary care service to all citizens of Ottawa, Ontario (estimated population in 2009: 885,715 inhabitants). It targets the population of high-risk mood disorder patients, namely, individuals with serious, complex, and/or rare mental disorders, who present multiple and complex needs, and whose treatment requirements cannot be met in the first line or at the more intensive levels of service. As such, these patients require more intensive and prolonged care resources. The study protocol was approved by the Research Ethics Board of ROMHC (Reference: REB#2006-22). A written informed consent was obtained from participants, where the rational of the study was explained and the participants gave consent to publish the results of the study, but kept their identity confidential.

The selection of the cases to be assessed by the ATC was as follows. A nurse examined all the referrals from community doctors (mostly family physicians) to the mood disorders program and chose the referrals which were the most likely to satisfy the criteria for the program, that is, subjects suffering from primary recurrent or chronic unipolar major depressive disorder or BD, were resistance to treatment, or severity required the intervention of the outpatient multidisciplinary team. About 20% of all referrals were included and assessed by the ATC team through a diagnostic and clinical assessment, so a standardized diagnosis was available only for these patients. The psychiatrists of the program assessed the remaining cases, making recommendations for treatment or following the patients as appropriate. From December 2006 to February 2009, the ATC assessed 409 subjects, who were referred to the program by physicians within the community; mostly family physicians. Of these subjects, 113 (39 men and 74 women) presented with primary diagnosis of BD type I or II.

### Diagnostic and clinical assessment

During the course of the initial visit, subjects underwent clinical interviews with a nurse, a social worker, an occupational therapist, a psychiatrist and a psychologist. Collection of information took on average 3 to 5 hours.

The Structured Clinical Interview for DSM-IV-TR (SCID) [35] was used to assess diagnosis on Axis I. The SCID screen was administered to all patients which included Mood and PTSD modules completed for every patient*.* Where anxiety, psychotic, or eating disorder was indicated based on SCID screening, these modules were administered as well. Substance abuse and dependence were flagged based on the chart review and the patient’s report during the interdisciplinary assessment. The correspondent module was administered where indicated.

In cases where a diagnosis of BD proved inconclusive with the use of SCID-I (“rule out diagnoses”), all efforts were made to produce a valid diagnosis, by consulting the charts and/or interviewing the treating psychiatrist. The psychotropic medications that were prescribed and their dosages were recorded by a nurse. The lithium levels were measured in the following month after the first assessment. A history of previous hospitalizations was also systematically collected by a nurse.

### Criteria: adherence to treatment guidelines

The CANMAT guidelines published in 2009 [18] were considered for purposes of evaluating treatment concordant with guidelines. CANMAT guidelines include first, second, and third line recommendations, where second and third line recommendations should be used when the first line are not effective, or not tolerated, or not indicated for other reasons. A systematic longitudinal pharmacological history of every patient was not available, so we could not verify if patients treated with second or third line treatments had already been prescribed with a treatment following a first line recommendation. However, our sample included patients referred to a tertiary care structure and the selection process of the sample included the criteria of having a resistant-to-treatment, or chronic, severe and highly recurrent disorder. In these cases, it is likely that the patients had already been prescribed with at least a first line treatment. For this reason, we considered second and third line treatments as concordant to guidelines.

Using the Structured Clinical Interview for DSM-IV (SCID) [35], we identified the nature of a given mood episode present in any month preceding the assessment (“current episode”). For cases exhibiting a current depressive episode, treatment was compared with recommendations for “acute bipolar depression” for BD type I (BD-I), as indicated in the Table four point three of the above publication [18] and BD-II (Table seven point two) [18]. For cases of hypomania, treatment was compared with recommendations for “acute mania” (Table three point three) [18]; otherwise, treatment was compared with recommendations for “maintenance treatment” for BD-I (Table five point five) and BD-II (7.4) [18]. No cases of acute mania were described among bipolar I patients at the moment of assessment.

Prescriptions for lithium, anticonvulsants, atypical antipsychotics, and antidepressants were considered for purposes of assessing prescribed treatments as concordant with the CANMAT guidelines, whether they were associated or not with benzodiazepines, zopiclone, or first generation neuroleptics. Although CANMAT guidelines do not explicitly specify the dose ranges for the recommended treatments, the recommendations are mainly based on clinical trials. Based on the results of these trials, we considered the following dosages to define a treatment as being in the “therapeutic range”, so following the CANMAT recommendations: lithium blood levels ≥0.5 mEq/L; valproate ≥ 750 mg daily; carbamazepine ≥600 mg daily; lamotrigine ≥ 50 mg daily; olanzapine ≥ 5 mg daily; risperidone ≥ 2 mg daily; quetiapine ≥150 mg daily.

The following exceptions were made: 1) trazodone at low dosage (≤50 mg) was not considered as “antidepressant”, given its use as a hypnotic; 2) amitryptyline at low dosage (<50 mg) was not considered an antidepressant, given its use as an anti-pain or hypnotic under low dosage. “Polypharmacy” was defined as the association of more than one mood stabilizer, atypical antipsychotic and antidepressant, where at least one of the prescribed treatments met the CANMAT recommendations, though in combinations not specifically recommended by the guidelines. The classification “doesn’t follow the guidelines” refers to patients not receiving any treatment included in the CANMAT recommendations.

### Data analysis

Data was analyzed using SPSS 19.0 statistical package (PASW, Chicago, IL). Pearson χ^2^ test and t-tests were used where appropriate. Bonferroni corrections were used in the analyses. We considered the following analyses: 1) in descriptive statistics, we compared the proportions of subjects taking at least one of the different classes of psychotropics at any dosage (lithium, anticonvulsants, antidepressants, benzodiazepines, neuroleptics, at least one psychotropic drug) by type of bipolar disorder (BD-I or BD-II) and nature of the mood episode (hypomanic, depressive, euthymia). Two-tailed tests were considered as significant when p < 0.001. 2) We then compared the proportions of subjects taking at least one mood stabilizer or atypical antipsychotic with a dosage in the therapeutic range, by type of BD and nature of the mood episode. Two-tailed tests were considered as significant when p < 0.001. 3) Finally, we compared the proportions of patients prescribed with guideline-concordant treatment for the following predictors: sex; age; previous hospitalizations; type of BD; nature of the current mood episode; comorbid anxiety disorder (current); comorbid substance use disorder (current); age at onset of the mood disorder. Two-tailed tests were considered as significant when p < 0.006. A logistic regression model was used to predict the independent likelihood of receiving treatment not concordant with guidelines.

## Results

### Characteristics of the sample

Sampling consisted of 32 patients diagnosed with BD- I, and 81 with BD-II. Sex and age were similar for both samples: two-thirds being female (N = 74, 65.5%); average age (SD) being 38.7 (11.2).

### Prevalence of psychotropic prescriptions by type of BD

All bipolar I patients and 90% of bipolar II patients were administered at least one psychotropic drug. Table 2 shows the number of patients receiving specific psychotropics, at any dosage. Statistical comparisons between BD-I and II were performed for classes of psychotropics only. Lithium was prescribed for one-third of the sample, more frequently in BD-I than BD-II. Among patients prescribed with lithium, a similar percentage of bipolar I and II patients were prescribed an adequate dosage, with serum levels ≥0.5 mEq/L (BD-I: N = 16 (80%); BD-II: N = 12 (80%). The median value of lithium serum levels were 0.59 and 0.79 in BD-I and II, respectively.

**Table 2:** Psychotropic treatment by type of bipolar disorder among patients attending an out–patient tertiary care service

	**Bipolar I disorder (N = 32)**	**Bipolar II disorder (N = 81)**	**Total**	**Daily dosage min–max**
	**N (%)**	**N (%)**	**N (%)**	**Mg**
**Lithium**	**20 (62.5)**	15 (18.5) ^*^	**35 (31.0)**	300–1950
**Anticonvulsants**	**10 (31.3)**	**36 (44.4) **^†^	**46 (40.7)**	
Valproate	3 (9.4)	14 (17.3)	17 (15)	250–2000
Carbamazepine	1 (3.1)	0	1 (0.9)	600
Lamotrigine	6 (18.8)	13 (16)	19 (16.8)	50–500
Gabapentin	1 (3.1)	6 (7.4)	7 (6.2)	300–1600
Topiramate	1 (3.1)	9 (11.1)	10 (8.8)	25–200
Levetiracetam	0	1 (1.2)	1 (0.9)	250
**Antidepressants**	**17 (53.1)**	**54 (66.7) **^†^	**71 (62.8)**	
SSRIs	7 (21.9)	27 (33.3)	34 (30.1)	
Citalopram	4 (12.5)	10 (12.3)	14 (12.4)	20–80
Escitalopram	1 (3.1)	12 (14.8)	13 (11.5)	5–40
Fluoxetine	1 (3.1)	1 (1.2)	2 (1.8)	20
Fluvoxamine	1 (3.1)	0	1 (0.9)	200
Paroxetine	0	2 (2.5)	2 (1.8)	40–60
Sertraline	0	2 (2.5)	2 (1.8)	100–200
SNRI	9 (28.1)	13 (16.0)	22 (19.5)	
Duloxetine	0	1 (1.2)	1 (0.9)	60
Venlafaxine	9 (28.1)	12 (14.8)	21 (18.6)	75–300
Moclobemide	0	1 (1.2)	1 (0.9)	600
Antidepressants Tricyclic	1 (3.1)	5 (6.2)	6 (5.3)	
Amitriptyline	1 (3.1)	4 (4.9)	5 (4.4)	10–100
Trimipramine	0	1 (1.2)	1 (0.9)	50
Other antidepressants	6 (18.8)	27 (33.3)	33 (29.2)	
Bupropion	2 (6.3)	13 (16.0)	15 (13.3)	100–450
Mirtazapine	0	4 (4.9)	4 (3.5)	7.5–60
Trazodone	5 (15.6)	13 (16.0)	18 (15.9)	50–200
**Anxiolytics benzodiazepines**	**10 (31.3)**	**16 (19.8) **^†^	**26 (23.0)**	
**Neuroleptics**	**18 (56.3)**	**33 (40.7) **^†^	**51 (45.1)**	
Neuroleptics first generation	2 (6.3)	4 (4.9)	6 (5.3)	
Methotrimeprazine	1 (3.1)	4 (4.9)	5 (4.4)	10–125
Trifluoperazine	1 (3.1)	0	1 (0.9)	3
Neuroleptics second generation	17 (53.1)	30 (37.0)	47 (41.6)	
Olanzapine	7 (21.9)	6 (7.4)	13 (11.5)	2.5–20
Quetiapine	8 (25.0)	18 (22.2)	26 (23.0)	12.5–450
Risperidone	2 (6.3)	8 (9.9)	10 (8.8)	0.5–2.00
Stimulants – methylphenidate	0	1 (1.2)	1 (0.9)	5
Hypnotics – Zopiclone	0	5 (6.2)	5 (4.4)	3.75–30
No psychotropics	0	8 (90.0)	8 (7.0)	

^*^ p < 0.001 ^†^ NS.

Anticonvulsants were prescribed for approximately 40% of the sample; more than 60% of bipolar patients were prescribed antidepressants. Second generation neuroleptics were prescribed much more frequently than first generation. Prescriptions for anticonvulsants, antidepressants, benzodiazepines and neuroleptics were not significantly different in the two types of BD. Bipolar I patients were more likely be prescribed with at least one mood stabilizer or atypical antipsychotic at therapeutic range than bipolar II patients (BD-I: 84% vs BD-II: 49%, χ^2^ = 11.6, df = 1, p < 0.001).

Our sample included only a minority of patients treated with one single mood stabilizer within monotherapy (lithium, lamotrigine, valproate, or carbamazepine), without being prescribed any antidepressants or second generation antipsychotics (bipolar I patients: N = 11 (34%); bipolar II patients: N = 12 (15%).

### Psychotropic medications administered by nature of mood episodes

No significant differences were found in the prescriptions by the nature of the mood episode, although there was a trend for subjects with a current depressive episode to be prescribed antidepressants more frequently than other subjects (72% vs. 50%, respectively, χ^2^ = 5.9, df = 1, p < 0.02) (Table 3). Especially noteworthy was the relatively frequent prescription of antidepressants to subjects suffering from current hypomanic episodes (43% in BD-I and 56% in BD-II).

**Table 3:** Classes of psychotropic treatment by type of bipolar disorder and nature of current mood episode

	**Bipolar I disorder (N = 32)**			**Bipolar II disorder (N = 81)**		
	**Hypomania**	**Depression**	**Euthymia**	**Hypomania**	**Depression**	**Euthymia**
	**N = 7**	**N** = **18**	**N** = **7**	**N** = **18**	**N** = **47**	**N** = **16**
	N (%)	N (%)	N (%)	N (%)	N (%)	N (%)
Lithium	4 (57.1)	11 (61.1)	5 (71.4)	3 (16.7)	8 (17.0)	4 (25.0)
Anticonvulsants	2 (28.6)	5 (27.8)	3 (42.9)	7 (38.9)	25 (53.2)	4 (25.0)
Antidepressants	3 (42.9)	13 (72.2)	1 (14.3)	10 (55.6)	34 (72.3)	10 (62.5)
Neuroleptics	3 (42.9)	11 (61.1)	4 (57.1)	7 (38.9)	20 (42.6)	6 (37.5)
First generation	0	1 (5.6)	1 (14.3)	1 (5.6)	3 (6.4)	0
Second generation	3 (42.9)	11 (61.1)	3 (42.9)	6 (33.3)	18 (38.3)	6 (37.5)
Benzodiazepines	0	7 (38.9)	3 (42.9)	3 (16.7)	8 (17.0)	5 (31.3)
At least one MS or AAP in therapeutic range*	7 (100)	15 (83.3)	5 (71)	8 (44.4)	26 (55.3)	6 (37.5)
At least one psychotropic drug	7 (100)	18 (100)	7 (100)	16 (88.9)	44 (93.6)	13 (81.3)

*MS or AAP in therapeutic range: lithium serum levels ≥0.5 mEq/L; valproate ≥750 mg daily; carbamazepine ≥600 mg daily; lamotrigine ≥50 mg daily; olanzapine ≥5 mg daily; risperidone ≥2 mg daily; quetiapine ≥150 mg daily.

### Prescriptions concordant with CANMAT guidelines

Eight-four patients (74.3%) were prescribed with at least one treatment congruent with CANMAT guidelines when considering only the type of the treatment. When we considered type and therapeutic dosage, the number decreased to 77 (68.1%) (Table 4). Among bipolar I patients, the number of patients prescribed with at least one treatment concordant with guidelines for both type and dosage was 24 (75%); 4 (12%) bipolar I patients were prescribed with at least one treatment concordant for type but not for dosage. The corresponding figures for BD-II were 53 (65.4%) and 3 (4%), respectively. Of the 47 bipolar II patients in a depressive phase, 12 patients were prescribed antidepressants within monotherapy, which is classified as a third line recommendation under the CANMAT guidelines.

**Table 4:** **Treatment concordant with CANMAT guidelines by medication and dosage in bipolar patients**, **by type of disorder and by nature of current episode**

	**Bipolar I disorder (N = 32)**			**Bipolar II disorder (N = 81)**			
	**Hypomania**	**Depression**	**Euthymia**	**Hypomania**	**Depression**	**Euthymia**	**Total**
	**N = 7**	**N = 18**	**N = 7**	**N = 18**	**N = 47**	**N = 16**	**N = 113**
**Follows guidelines strictly**	**4** (**57**.**1%**)	**8** (**44**.**4%**)	**4** (**57**.**1%**)	**3** (**16**.**7%**)	**29** (**61**.**7%**)	**4** (**25**.**0%**)	**52** (**46**.**0%**)
First line treatment	4 (57.1%)	5 (27.8%)	3 (42.9%)	3 (16.7%)	0	1 (6.3%)	16 (14.2%)
Second line treatment	0	1 (5.6%)	1 (14.3%)	0	14 (29.8%)	3 (18.8%)	19 (16.8%)
Third line treatment	0	2 (11.1%)	0	0	15 (31.9%)	0	17 (15.0%)
**Polypharmacy with at least one treatment recommended in guidelines**	**0**	**7** (**38**.**9%**)	**1** (**14**.**3%**)	**1** (**5**.**6%**)	**14** (**29**.**8%**)	**2** (**12**.**5%**)	**25** (**22**.**1%**)
**No treatment following the guidelines**	**3** (**42**.**9%**)	**3** (**16**.**7%**)	**2** (**28**.**6%**)	**14** (**77**.**8%**)	**4** (**8**.**5%**)	**10** (**62**.**5%**)	**36** (**31**.**9%**)

*Treatment concordant for type and dosage of prescribed mood stabilizers.

The reasons for non-concordance with guidelines appeared to vary according to the nature of the episode (Table 5). A high percentage of subjects with current hypomanic episodes were prescribed with no treatment concordant with guidelines (17 out of 25 patients, 68%); in 13 of these 17 cases, subjects were prescribed antidepressants. Ten bipolar II patients in a hypomanic phase were prescribed with antidepressants. Six of these patients had “mixed” depressive-hypomanic symptoms corresponding to a “dysphoric” state, and two patients presented rapid cycling. The remaining two patients had mild to moderate hypomanic symptoms. Among subjects for whom treatment non-concordant with guidelines was prescribed, a total of 11 patients were prescribed antidepressants without any mood stabilizer.

**Table 5:** **Reasons for non**-**concordance of treatment with guidelines**

	**Bipolar I disorder with no treatment concordant with guidelines (N = 8)**			**Bipolar II disorder with no treatment concordant with guidelines (N = 28)**			
	**Hypomania**	**Depression**	**Euthymia**	**Hypomania**	**Depression**	**Euthymia**	**Total**
	**N = 3**	**N = 3**	**N = 2**	**N = 14**	**N = 4**	**N = 10**	**N = 36**
No psychotropics	0	0	0	2	3	3	8 (22.2%)
Antidepressants without any mood stabilizer	0	1	0	5	-	5	11 (30.6%)
Antidepressants in hypomania	3	-	-	5	-	-	8 (22.2%)
Low dosage of treatment	0	2	2	1	1	1	7 (19.4%)
Other prescriptions of psychotropics, not following guidelines	0	0	0	1	0	1	2 (5.6%)

### Factors associated with the prescription of at least one treatment concordant with guidelines by type and dosage of the medication

The associations between several clinical factors and treatment concordant with guidelines (Table 6) were studied. Univariate analyses showed that hypomanic patients presented poorer guideline concordance (p < 0.001). There was also a similar non significant trend for patients having had no previous hospitalizations (p < 0.05) and presenting a current substance disorder (p < 0.06).

**Table 6:** **Number** (%) **of subjects receiving at least one treatment following CANMAT guidelines by type and dosage of medication**, **by clinical and sociodemographic factors**

**Variable**	**At least one treatment concordant with guidelines N (%)**	**N**	**Degree of freedom**	**Χ**^**2**^	**P**
Sex					
Man	26 (66.7%)	39	1	0.06	0.81
Woman	51 (68.9%)	74			
Previous hospitalizations					
Yes	41 (77.4%)	53	1	3.91	0.05
No	36 (60.0%)	60			
Type of bipolar disorder					
Type I	24 (75.0%)	32	1	0.97	0.32
Type II	53 (65.4%)	81			
Nature of the current mood episode					
Hypomanic	8 (32.0%)	25			
Depressive	58 (89.2%)	65	2	32.7	0.001
Euthymic	11 (47.8%)	23			
Comorbid anxiety disorder (current)					
Present	19 (67.9%)	28	1	0.001	0.97
Absent	58 (68.2%)	85			
Comorbid substance use disorder (current)					
Present	15 (53.6%)	28	1	3.64	0.06
Absent	62 (72.9%)	85			
**Variable**	**Mean** (**SD**)	**N**		**t**-**test**	**P**
Age (mean ± SD)					
Treatment – concordant	39.8 ± 11.3	77	1	1.58	0.18
Treatment – not concordant	36.3 ± 10.7	36			
Age at onset (mean ± SD)					
Treatment – concordant	22.4 ± 8.0	77	1	1.72	0.09
Treatment – not concordant	25.5 ± 9.0	36			

To test the independent association of the same variables with a poorer concordance of treatment to guidelines, we performed a logistic regression, where concordant treatment with guidelines was the dependent variable (0 = treatment concordant, 1 = treatment non concordant). Previous hospitalizations, age at onset, current anxiety disorder, current substance use disorder, type of BD (type I = 0, type II = 1) and nature of the current episode (depressive or euthymia = 0; hypomanic = 1) were entered as independent variables. Analysis was adjusted for sex and age. The nature of the episode was treated as a dichotomic variable, because of the small numbers. Actual age and age at onset were entered as continuous variables. Only the nature of the mood episode was independently associated with treatment not concordant to guidelines, with hypomania predicting poorer concordance (hypomania vs. depressive and euthymic: OR (95% CI) = 7.34 (2.6 to 20.8), p < 0.001).

## Discussion

### Prevalence of psychotropic treatment

Only 7% of the patients were treatment-free; antidepressants were the most prescribed treatment, especially in BD-II; lithium was administered to 62% of the bipolar I patients and only 18% of the bipolar II patients.

In comparing the findings of our study with the results from scientific literature, we noticed that the percentage of patients not given any psychotropics in our sample was similar to results taken from previous studies conducted within academic centres or teaching hospitals where percentages ranged from 2% to 8% [36-39] were noted. Verdoux et al. [40] found lower percentages (0.9%) in hospital settings. Higher percentages (ranging between 16% and 22%) were found by Blanco et al. [41], examining data on a nationally representative group of visits to psychiatrists in an office-based practice within the US.

In our study, the global percentage of patients given lithium was 31%, which is in the low range of percentages found in other studies, ranging from 30% to 53% [36-38,40-46]. This low rate is comparable with that observed in the United States where the percentage of bipolar patients prescribed with lithium has decreased from 50.9% in 1992–95 to 30.1% in 1996–99, based on the National Ambulatory Medical care survey [41]. By contrast, European countries show a higher percentage of lithium use compared with the United States [36-38,40,42-46]. Decrease in lithium use has paralleled the increased use of valproate (from 10.9% to 26.6%) [41], and of second generation antipsychotics, (from 1.2% to 17.0% in Blanco et al. [41]; see also Hayes et al. [47] Pillarella et al. [48]). The low percentage of lithium use found in our study may well reflect the general North-American trend. Our study actually indicates both second generation antipsychotic and valproate use rates as being relatively high (41.6% and 15.0%, respectively), and this was consistent with the increasing trends in the use of such medications over the past decade.

In conducting our study, the type of BD appeared to be an important factor in predicting the use of lithium, with BD-I recording a much higher proportion of lithium users. This result is at odds with the STEP-BD data [46] which indicates a similar percentage of lithium users in BD-I and II (39.4% and 35.7%, respectively). On the other hand, Arvilommi et al. (2007) found that bipolar I patients were prescribed with mood stabilizers more often than bipolar II patients, which is consistent with our results, where the percentage of bipolar I patients taking at least one mood stabilizer or atypical antipsychotic in the therapeutic dosage was almost double than in bipolar II patients. This might not only reflect the CANMAT guidelines, but also the perceived lower degree of severity in BD-II and low consistency diagnosis. In our sample, about 20% of patients taking lithium had levels below the therapeutic range, which is consistent with the results of previous studies [30,36].

The percentage of bipolar patients given antidepressants in our study (62.8%) was similar to that found in similar studies conducted in North America. In the United States studies, corresponding percentages range from 40% to 74% [41,44-46,49,50]. Studies by Blanco et al. [41] indicate a constant pattern in the use of antidepressants between 1992–1995 and 1996–1999 (45.8% and 45.7%, respectively). As expected, bipolar patients with depressive episodes seemed to be more likely to take antidepressants than other bipolar patients in our study. A similar trend was observed in the STEP-BD study [46].

Interestingly, there is also a transatlantic difference in antidepressant use, more frequent in the United States than in Europe [42] In UK studies, the percentage of antidepressant use varies from 14.3% to 38% [36-38,43], whereas in France, Verdoux [40] et al. found 34.2% of bipolar patients prescribed with antidepressants.

In our sample, no bipolar patients were prescribed aripiprazole or ziprasidone, although CANMAT guidelines specifically recommended the two atypical antipsychotics in the treatment of BD-I since 2005 [16]. This can be due to the fact that Health Canada approved their use in the treatment of bipolar disorder only in 2009.

In our sampling, monotherapy with mood stabilizers was not a frequent approach, consistent with literature on the subject where only a minority of bipolar patients benefited from monotherapy treatment with lithium or other mood stabilizer (Ahmed and Anderson [36]: 23%; Frangou et al. [43]: 23.8%; Lloyd et al. [38]: 29%; Ghaemi et al. [46]: 10.8%; Verdoux et al. [40]: 14%).

In recent years, polytherapy has been used more frequently in clinical practice [44,51]. Polytherapy is more commonly administered for depressed bipolar patients where monotherapy is much less efficacious [52], while guidelines for acute mania recommend monotherapy with mood stabilizers or antipsychotics. The high frequency of polytherapy noted in our study may well reflect the higher prevalence of depression; it also suggests that the severe state of patients referred to our tertiary care services are more likely to have comorbidities or disorders resistant to treatment.

### Concordance with treatment guidelines

Treatment was congruent with guidelines in 68% of our sampling cases, when both type and dosage of the medications were considered. This percentage was quite high when compared with other studies. The larger spectrum of recommendations present in the CANMAT guidelines, compared with other guidelines, can partially explain this result [53].

Divergence from guidelines was mostly noted for patients suffering from hypomania, with 68% of hypomanic patients receiving non-concordant treatment to guidelines. In more than half of the cases, treatments not congruent with guidelines were due to inappropriate treatments with antidepressants; the remaining cases of non-concordance were due to the absence of psychotropic treatment or to inadequate dosage. Also noteworthy is that more than one-third of depressed bipolar II patients receiving treatment concordant with guidelines were prescribed antidepressants within monotherapy, which constitutes a third line recommendation under the CANMAT guidelines, and almost one third of all bipolar II patients were treated with antidepressant without any mood stabilizer.

The use of antidepressants for bipolar depression is still controversial. The major criticism regarding use of antidepressants in instances of bipolar depression is based on the risk of destabilizing mood, switching into mania or hypomania [54-56] or increasing the cycle frequency [57,58]. Such negative effects appear to be limited to certain classes of antidepressants [59-61]. Factors which appear to increase the risk of destabilization are: presence of mixed states [62,63], subthreshold manic symptoms at baseline [64] and BD-I (versus BD-II) [65,66], which suggests that different subpopulations may present different vulnerabilities to affective switching. In the STEP-BD study, approximately 15% of bipolar patients receiving long-term treatment with antidepressants developed a chronic irritable dysphoric state (ACID syndrome). The study indicated that the risk of developing ACID symptoms was linked to a previous history of antidepressant-induced affective switch, and to female gender [67]. Finally, the use of antidepressants may significantly increase the risk of suicidal ideation [68].

The efficacy of antidepressants in the acute treatment of bipolar depression is uncertain. The STEP-BD study showed similar efficacy comparing lithium or divalproex, alone or associated with an antidepressant [61]. In his meta-analysis, Gijsman et al. [69] suggested antidepressants as being effective in the short-term treatment of bipolar depression, but in a more recent meta-analysis, antidepressants were found not to be superior in any way to placebos in the acute treatment of bipolar depression [70]. Then again, Altshuler et al. [71] suggested the usefulness of maintaining antidepressant treatments within the subpopulation of patients who require both a mood stabilizer and an antidepressant to achieve complete remission from depressive episodes. Also controversial is the efficacy of long-term treatment with antidepressants in protecting patients from the recurrences of bipolar depression [55,72]. A meta-analysis of seven trials involving long-term treatments concluded that long-term adjunctive antidepressant treatments were not superior to mood stabilizers alone in protecting patients from recurrences, and that antidepressant monotherapies were associated with higher risks of mania or hypomania relapses [73].

Antidepressants administered within monotherapy are contraindicated for BD-I, because of the high risk of inducing mania or rapid cycling [54]. However, the risk/benefits may be different in BD-I and BD-II, and some studies suggest the efficacy and safety of antidepressant monotherapies in the treatment of BD-II [74-77]. CANMAT guidelines recommend antidepressants associated with mood stabilizers or atypical antipsychotics in the treatment of acute bipolar depression. Antidepressant monotherapy is only recommended as a third line treatment for BD-II, particularly for those with infrequent hypomanias [18].

Finally, though there is agreement on the different guidelines in the discontinuance of treatment with antidepressants during manic episodes, nevertheless they are at times prescribed in cases of acute mania in clinical practice [33,78]. Within our sampling, antidepressants were prescribed to certain bipolar II patients suffering from current hypomanic symptoms with “mixed” depressive symptoms or with rapid cycling. In both of these conditions, antidepressant treatment is specifically contraindicated [63].

Non-concordant treatment with guidelines observed in our sample can be explained in different ways. First, the diagnosis of BD, especially type II, can be difficult at the onset of the disorder. In most cases, BD-II patients consult their doctor during the depressive phase, not during the hypomanic phase. Identifying the hypomanic episodes in BD-II can be difficult if appropriate questions are not asked, or if family members are not properly interviewed. Thus, before it is finally recognized, BD can be diagnosed as a recurrent depression over several years [79-82]. Some of the patients in our sample were misdiagnosed as having recurrent major depressive disorder. The second possible reason is that treatment with antidepressants, administered during episodes of depression or for purposes of preventing further episodes, is not suspended during a hypomanic phase, either because patients do not require medical intervention or because symptoms are not recognized. There is no consensus in the current guidelines on criteria governing time frames necessary to interrupt antidepressants [53], which can create confusion as to the appropriate discontinuation of antidepressants following depressive episodes. It is noteworthy that the CANMAT guidelines do not make explicit recommendations for BD-II hypomania, leaving some potential confusion for the reader whether recommendations for acute mania have to be adopted. Patients not adhering to treatment with mood stabilizers [83-85] is a third possible factor which explains the frequency of treatment with antidepressants, especially in monotherapy. Finally, there remains the possibility of general practitioners not following guidelines for lack of awareness, lack of agreement, or lack of familiarity.

Although the study focused on patients seen between the end of 2006 and beginning of 2009, we decided to use the 2009 guidelines rather than 2007, as some of the recommendations adopted in 2009 were already suggested in the previous version. For example, the possibility to use antidepressant monotherapy in bipolar II patients in depressive phase was already described in 2007, and more explicitly formulated in 2009. Other new recommendations adopted in the 2009 CANMAT version, which could potentially affect the percentage of concordance with guidelines in our sample, concerned with the use of quetiapine monotherapy as first line recommendation of BD-II depression and in BD-I maintenance; the use of divalproex monotherapy as second line recommendation in BD-I and BD-II depression; and the use of quetiapine in combination with lithium or divalproex in BD-I maintenance. In our sample, only two treatments would have been classified in a different way if we had adopted the 2007 guidelines: two BD-II depressive patients taking divalproex monotherapy would have been classified as “doesn’t follow guidelines”, instead of “second line treatment”.

Our study, however, dealt with limitations. Our sample was relatively small, most notably as far as stratification was concerned. Also, we had to contend with a strong representation of depressed patients, which corresponds to a higher prevalence of depressive symptoms in the bipolar population.

## Conclusions

In conclusion, the actual results support the efficacy of efforts made over the past years in attempting to communicate CANMAT guidelines. Our studies indicate a relatively high rate of concordance to guideline treatment by general practitioners. A significant caveat was identified, however, in the use of antidepressant medications, especially with respect to treatment of bipolar patients with hypomanic symptoms. Our study identifies the inappropriate use of antidepressants as one of the factors in outlining non-concordant treatment with guidelines. Although there is no evidence supporting the widespread use of antidepressants in BD, and moreover, that there be evidence supporting the use of other agents in its treatment, antidepressants remain the most frequently prescribed class of psychotropic medications in this regard. In the absence of across-the-board consensus on the use of antidepressants in treating BD, physicians have to individualize the risk/benefits for each patient, and repeatedly reassess and re-evaluate the need for antidepressants, which makes the treatment of bipolar patients particularly challenging. Given the high prevalence of depressive symptoms in bipolar patients, it appears that the symptoms, rather than the disorder, are often the target for treatment. Future research and intervention can change the current practice [86]. A recent study [87] has shown that educational programs were the best means of increasing the likelihood of general practitioners correctly assigning a subtype diagnosis in the treatment of BD, and prescribing mood stabilizers instead of antidepressants.

## Abbreviations

AD: Antidepressant; AAP: Atypical antipsychotic; APA: American psychiatric association; BD: Bipolar disorder; CANMAT: Canadian network for mood and anxiety treatments; DSM-IVTR: Diagnostic standardized manual - revised; MS: Mood stabilizer; ROMHC: Royal Ottawa mental health centre; SCID: Structured clinical interview for DSM-IV-TR; STEP-BD study: Systematic treatment enhancement program for bipolar disorder.

## Competing interests

Both authors declare that they have no competing interests.

## Authors’ contributions

SP contributed to design, analysis and interpretation of data and drafting the manuscript. Dr. JCB contributed to conception, design, interpretation of data and revising the manuscript for important intellectual content. Both authors have given final approval of the version to be published.

## Pre-publication history

The pre-publication history for this paper can be accessed here:

http://www.biomedcentral.com/1471-244X/13/211/prepub
